# Supplementary material for: Emerging practices supporting diabetes self-management among food insecure adults and families: A scoping review
Source: PLoS One. 2019 Nov 6;14(11):e0223998. doi: 10.1371/journal.pone.0223998 (PMC6834117; doi:10.1371/journal.pone.0223998)
Supplement: Attachment — Submitted filename: Response to Reviewers.docx [file pone.0223998.s003.docx]

We would like to thank the editor for their thoughtful and constructive feedback and the opportunity to strengthen the manuscript and resubmit our manuscript. Please find below our responses to the editor’s comments. Thank you.

Dear Enza Gucciardi and co-authors,
I appreciate the efforts made to improve the paper and I believe the reviewers' suggestions were a great contribution. However, I understand and agree with the comments presented by them regarding the limitation to studies in the United States and Canada. Inlcuing this issue in the limitation seems appropriate, but my concern is that such statement is just a little comment at the end of the paper that will be imperceptible for most readers. I have the conviction this paper will have a global impact and maybe missinterpreted by readers from low-and-middle income countries, and your manuscript does not include evidence from these countries. There are two decisions made by the authors that, in my perspective, are the main drivers of this limitation:
1. Studies written in English only: at least in South America, there is a vast literature on this topic that does not have the aim to achieve international journals and are written in Portuguesse or Spaninsh (studies from Brazil, Mexico, Chile representing a total of 350 million inhabitants - as big as Canada and United States together) and published in journals from these countries as their objective is to provide evidence at a national level that may influence health and nutritional policies. Once the large amount of morbidity and deaths due to NCDs such as diabetes AND food insecurity is concentrated in low-and-middle income countries (it can be as high as 87% - see <http://www.scielo.br/pdf/csc/v22n2/1413-8123-csc-22-02-0637.pdf>), the decision of focusing on papers published in English only is a huge limitation of the study that should be worth mentioning in the abstract and conclusion as well. Therefore, although further research is nedded as stated in both sections of the manuscript, you can not affirm there is "lack of studies, especially outside
North America". These studies exist, but your study was unable to identify them because of the search strategy. For example, if you include the latinoamerican base LILACS (<http://lilacs.bvsalud.org/en/>) and use the terms "autocuidado diabetes" you would find 450 papers in the topic of diabetes self-management. As I mentioned before, most low-and-middle income countries live in food insecurity. Thus, using that term would exclude a good proportion of papers that would fit your paper aims.

Thank you for your thoughtful comment and we agree to add more context given the limitation of our search strategy for not including non-English studies. This was added in our abstract and limitation section as suggested. Please see the track changes in the manuscript and text below:

Abstract

“A major limitation of this review is the lack of global representation considering no studies outside of North America satisfied our inclusion criteria, due in part to the English language restriction.”

Limitation section

“The lack of identified studies outside North America can be partly attributed to the English language inclusion criteria”.

2. As stated in one of your answers to the reviewers "The second paper mentioned above was used in our discussion but was not eligible in our review as it included people with
various chronic diseases, in addition to diabetes. We limited our review to those with diabetes." International studies have shwon that around 30-60% of adults are affected by two or more chronic conditions (prevalence increases with age). Therefore, excluding studies evaluating sample of patients affected by diabetes only would be making your findings less generalizable and probably reflecting strategies targeting younger age grous. The table with the main findings does not include details on the age of the participants to evaluate this.

I am not suggesting that your group reasseses the methods or re-write the paper entirely. I am just requesting a better statement in the abstract and discussion section that the lack of studies outside North America does not mean there are not studies in other countries, but was a limitation imposed by the research strategy (i.e. English only and restricted to diabetes but no other conditions).

Otherwise, this paper is an excellent contribution to the scientific literature.

Response: We hope we have addressed your concern with the added statements in the abstract and limitation section regarding studies outside of North America. We understand the major limitation of our paper.

Our goal was to be more specific to inform diabetes practice as diabetes is a complex set of self-management protocols including food (beyond healthy eating, but consistent carbohydrate load throughout the day) medications to take or not take, to name a few, which may not be applicable to other chronic conditions. Given practice guidelines provide information based on disease state we wanted to supplement practice guidelines with emerging recommendations for those challenged by food insecurity.
